# Determinants of health-related quality of life proxy rating disagreement between caregivers of children with cancer

**DOI:** 10.1007/s11136-019-02365-9

**Published:** 2019-12-09

**Authors:** Niki Rensen, Lindsay M. H. Steur, Sasja A. Schepers, Johannes H. M. Merks, Annette C. Moll, Gertjan J. L. Kaspers, Raphaële R. L. Van Litsenburg, Martha A. Grootenhuis

**Affiliations:** 1 Pediatric Oncology, Emma Children’s Hospital, Amsterdam UMC, Cancer Center Amsterdam, Vrije Universiteit, Amsterdam, The Netherlands; 2 Princess Máxima Center for Pediatric Oncology, Utrecht, The Netherlands; 3 Psychosocial Department, Emma Children’s Hospital, Amsterdam UMC, University of Amsterdam, Amsterdam, The Netherlands; 4 Pediatric Oncology, Emma Children’s Hospital, Amsterdam UMC, University of Amsterdam, Amsterdam, The Netherlands; 5 Department of Ophthalmology, Amsterdam UMC, Cancer Center Amsterdam, Vrije Universiteit, Amsterdam, The Netherlands

**Keywords:** Patient-reported outcomes, Proxy reports, Quality of life, Parents, Child, Cancer, Oncology

## Abstract

**Purpose:**

Proxy reports of health-related quality of life (HRQoL) are commonly used in pediatric oncology. However, it is not known if caregivers’ reports differ. This study therefore aims to compare paternal and maternal proxy reports, and explore determinants of couple disagreement (sociodemographic and medical characteristics, and parental QoL and distress).

**Methods:**

Both parents completed the PedsQL generic (child’s HRQoL), Short Form-12 (own QoL) and Distress Thermometer for Parents. To assess agreement in child HRQoL, intra-class correlation coefficients (ICCs) were calculated. Differences between fathers/mothers were assessed with paired *t* tests. Systematic disagreement patterns were visualized with Bland–Altman plots. Characteristics of parental couples with a mean proxy difference in the highest quartile (highest proxy score minus lowest proxy score) were explored with multiple logistic regression analysis.

**Results:**

Parents of 120 children with cancer (87% post-treatment, mean age 11.0 ± 5.7 years) participated. No significant differences were found between paternal and maternal proxy scores, and agreement was good on all scales (ICCs 0.65–0.83). Bland–Altman plots revealed no systematic disagreement patterns, but there was a wide range in magnitude of the differences, and differences went in both directions. Couples with a mean proxy difference (irrespective of which direction) in the highest quartile (± 20 points) were more likely to have a child in active treatment, with retinoblastoma or relapsed disease, and to diverge in their own QoL.

**Conclusions:**

If proxy reports of only one parent are available, clinicians may reasonably assume that paternal and maternal reports are interchangeable. However, if in doubt, respondent’s sex is not of major importance, but clinicians should be aware of patient’s and family’s characteristics.

## Introduction

Parent proxy reports are commonly used in pediatric care and research [1]. Although differences between child and parent proxy reports are clearly present when both are available [2–5], proxy reports are important for several reasons. Most importantly, proxy reports are indispensable when children are neurocognitively impaired, too young, or too ill to respond for themselves [6, 7]. This is often true in childhood cancer. As a result of improvements in treatment and survival, patient-reported outcomes (PROs) such as health-related quality of life (HRQoL) are becoming increasingly important in this field. Extensive research has shown that children are at risk for HRQoL impairment, both during and after treatment. This is reported by children as well as their parents, although children tend to be more positive about their own functioning [4, 8–11].

In clinical practice and studies where parent proxy respondents are used, it is customary to ask just one parent to report on the child, most often the mother [8, 9]. However, it is not well known if there are systematic differences between caregivers in their proxy HRQoL ratings [3, 4]. A large study in parents of healthy Iranian children concluded that paternal and maternal proxy HRQoL reports were interchangeable, although there were small but statistically significant differences between parents [12]. However, this is only one study. Furthermore, results in parents of healthy children may not be directly applicable to ill children, since circumstances are different and parents of ill children face additional distressing challenges [13, 14].

Factors that are known to influence parent proxy ratings in (chronically) ill children are among others child’s age and sex, parental educational level and cultural background, and the parent’s own health, QoL and distress [3, 4, 15–19]. It is not known, however, to what extent these factors play a role in disagreement in parent proxy ratings between mothers and fathers of (chronically) ill children. Since previous research in parents of children with cancer has shown that, within one family, mothers’ self-reported QoL is lower than fathers’, we hypothesize that mothers report lower HRQoL for their child as well [14]. Moreover, medical factors such as the child’s treatment status, time since diagnosis and type of diagnosis might be of influence on proxy ratings, since these factors entail different stressors [19].

It is important to explore potential disagreement in proxy ratings between caregivers, because the presence of these differences could influence the interpretation of parent-reported outcomes in pediatrics. Additionally, it is important to identify which parental couples are more likely to diverge in their proxy scores, in order to better understand in which situations more caution is warranted regarding the interpretation of these outcomes.

This study therefore primarily aims to compare paternal and maternal proxy reports of children’s HRQoL in a large cohort of current and former pediatric cancer patients (hereafter referred to as children with cancer). Second, this study aims to explore possible determinants of couple disagreement in this population (e.g. sociodemographic and medical characteristics, and parents’ own reported QoL and distress).

## Methods

### Study population

Data of this study are derived from the “Amsterdam Parent Project”, a cross-sectional multicenter study on parental sleep, QoL and psychosocial functioning in childhood cancer. A detailed description of the Amsterdam Parent Project is given elsewhere [14]. For the purposes of the current study, the questionnaires on parent-rated child HRQoL as well as those on parental QoL and distress were included. In the Amsterdam Parent Project, both parents were invited through mail to complete validated questionnaires independently from each other, either on paper or through an online assessment. If parents were not living at the same address, the parent that received the invitation was asked to transfer it to the other parent. Inclusion criteria were having a child diagnosed with cancer or any type of brain tumor between January 2010 and January 2015 in the Emma Children’s Hospital, Amsterdam University Medical Centers (location VUmc or AMC) and receiving follow-up care in one of these centers at time of study. Parents of deceased children or children receiving palliative care were excluded, as well as parents who were insufficiently fluent in Dutch to complete questionnaires independently.

Ultimately, 352 parents (202 mothers and 150 fathers, response rate 48%) were included, comprising 121 parental couples. One couple was excluded from the current study because they had a child younger than 2 years of age and therefore could not complete the proxy HRQoL questionnaire (see below). The data of the remaining 120 couples are reported here.

### Measures

#### Sociodemographic and medical variables

The following sociodemographic characteristics were assessed with a general survey: parental age and sex; highest attained educational level (according to Statistics Netherlands [20]; dichotomized for analysis as low-middle vs high); and country of birth (The Netherlands vs other). Moreover, the following child variables were extracted from the children’s medical files: age and sex; diagnosis type (hematologic malignancy, brain tumor, solid tumor, retinoblastoma); cumulative treatment, defined as low risk (no intervention, surgery only, local therapy other than radiation), middle risk (chemotherapy with or without surgery) or high risk (any combination with radiation and/or stem cell transplant); relapse or second tumor (yes or no); time since diagnosis; and active treatment at time of study (yes or no).

#### Child HRQoL

In parents of children aged two years and over, child HRQoL was assessed with the parent proxy version of the Pediatric Quality of Life InventoryTM (PedsQL) 4.0 Generic Core Scales (2–18 years) [21]. The PedsQL consists of 23-items, addressing four domains: physical, emotional, social and school functioning. The sum of the scale scores is used to calculate a mean total score. Moreover, two summary scores can be calculated: the Physical Health summary score (8 items) and the Psychosocial Health Summary Score (PSHS, 15 items) [22]. Scores range from zero to 100 and higher scores indicate better HRQoL. Missing values were handled according to the scoring guidelines, which means that scale scores were calculated by imputing the mean of the completed items in that scale, if at least 50% of the scale items were completed [21]. The Dutch version of the PedsQL 4.0 Generic Core Scales has adequate psychometric properties. Norm values collected by Varni et al. (proxy reports 2–18 years) are available [21]. Cronbach’s alpha in the study population ranged from 0.71 (social domain score) to 0.92 (physical domain and total score).

#### Parental QoL

Parental QoL was assessed with the Short Form-12 (SF-12), a generic QoL instrument for adults [23]. It measures functional health and well-being by means of two summary scores: the Physical Component Summary score (PCS) and Mental Component Summary score (MCS). The MCS and PCS are norm-based standardized summary scores with a mean of 50 and standard deviation (SD) of 10 in the general US population [23]. Higher scores indicate better QoL. Missing values were not imputed. The Dutch version has adequate validity and reliability and Dutch norm values are available [24].

#### Parental distress

To measure parental distress, the thermometer score of the Distress Thermometer for Parents (DT-P) was used. Parents were asked to rate their overall distress from 0 to 10, with a score of 4 or higher indicating clinical distress levels. This cut-off has been previously validated in parents of healthy and chronically ill children [25, 26].

### Statistical analysis

#### Study population

Differences in child’s demographic and medical characteristics between the 120 children in the current study and the other children of the Amsterdam Parent Project (*n* = 111) plus non-responders (*n* = 247) were calculated by using independent *t* tests for continuous variables and Chi square tests for dichotomous and categorical variables.

#### Description of proxy-reported child HRQoL, parental QoL and distress

For the description of proxy-rated child HRQoL and parent’s own QoL, means and SD were calculated for each scale, separately for fathers and mothers. Mean proxy scores of fathers and mothers of children with cancer were compared to proxy scores of parents of healthy children [21] using one-sided *t* tests. Fathers’ and mothers’ own QoL and distress scores were compared with paired *t* tests. For both differences with parents of healthy children and difference between fathers and mothers, significance level was set at *p* < 0.008 after adjusting for multiple testing (Bonferroni, 0.05/6).

Relationships between parental proxy scores and own QoL and distress were assessed with Pearson’s correlations, separately for fathers and mothers. Correlations between 0.2 and 0.5 were considered small, 0.5–0.8 moderate, and ≥ 0.8 strong.

#### Agreement in paternal and maternal proxy HRQoL scores

To assess agreement in paternal and maternal proxy scores, intra-class correlation coefficients (ICCs) with confidence intervals were calculated. A two-way mixed effects model was used (single measures, absolute agreement) [27]. ICCs of < 0.40 were considered poor, 0.40–0.60 fair, 0.60–0.80 good and ≥ 0.80 excellent.

Additionally, the mean differences between paternal and maternal scores were analyzed with paired *t* tests. The meaning of the differences was displayed as effect sizes, Cohen’s *d* (mean (*a*) − mean (*b*), divided by the pooled SD of both groups). Effect sizes of 0.2–0.5 were considered small, 0.5–0.8 moderate and ≥ 0.8 large [28].

Finally, Bland–Altman plots were constructed for each scale to reveal any systematic patterns in disagreement between parents [29]. With this technique, for each couple, the mean of the father’s and mother’s score is plotted on the *x*-axis against the mean difference between the both parents (father’s score minus mother’s score) on the *y*-axis. Additionally, a horizontal reference line is added at the mean difference between fathers and mothers of the entire sample, and limits of agreements are added as horizontal reference lines at ± 2SD of this mean difference. Ideally, the mean difference line intersects zero at the *y*-axis, and all couples’ observations are around this line.

#### Determinants of caregiver disagreement

For each outcome, multiple logistic regression models were built to assess predictors of a mean proxy difference in the highest quartile (p75-100) compared to the lowest three quartiles (p0-75). For this purpose, quartile groups were created of the overall mean proxy difference, irrespective of the direction of this difference (i.e. highest proxy score minus the lowest proxy score in each couple, instead of father’s score minus mother’s score). This was done because the Bland–Altman plots did not show systematic patterns in the differences, but yet a wide range in the magnitude of the differences, and differences went in both directions (equally in favor of fathers and mothers).

The above-mentioned child (medical) variables were assessed. Furthermore, the following characteristics of parental couples were assessed: difference in parental mental QoL (MCS, highest score minus lowest score), discrepancy in parental distress score (one parent with and the other parent without clinical distress; yes or no), difference in educational level (yes or no), difference in cultural background (yes or no).

A backward regression with preselection was performed. First, the relationship of all variables with the outcome were univariately tested. Variables with a statistically significant relationship, defined as *p* value < 0.15, were retained. Second, these variables were tested in a multiple model and only variables significantly associated with the outcome (*p* value set at < 0.10) were retained in the final model. Effects were displayed as odds ratios (OR) with 95% confidence intervals.

All analyses were done with IBM SPSS Statistics version 22.0 and if not otherwisely specified above, a two-sided* p* value of 0.05 was considered as statistically significant.

## Results

### Study population

The 120 parental couples in this study consisted of 120 mothers and 120 fathers; there were no same-sex couples. Three respondents were step parents (two stepfathers and one stepmother). Mean parental age was 43.6 (± 7.6) years (45.1 (± 7.6) in fathers and 42.2 (± 7.4) in mothers). Their children (*n* = 120) were on average 3.3 (± 1.4) years from diagnosis and the majority (87%) had finished treatment. No significant differences were found in child characteristics between participants of the study and other children of the Amsterdam Parent Project plus non-responders, except for the percentage of children in active treatment (13% in participants vs 7%, *p* = 0.04; data not shown). Parent and child characteristics are summarized in Table 1.

**Table 1:** Parent (*n* = 240) and child (*n* = 120) characteristics

	Parents	
	Mothers	Fathers
Relationship to child		
Parent	119	118
Stepparent	1	2
Mean age (SD)	42.2 (7.4)	45.1 (7.6)
Marital status (%)		
Married/living together	113 (94.2)	114 (95.0)
Single/divorced/widowed	7 (5.8)	6 (5.0)
Country of birth (%)		
The Netherlands	112 (93.3)	110 (91.7)
Other	8 (6.7)	10 (8.3)
Educational level^a^ (%)		
Low	11 (9.2)	13 (10.8)
Middle	61 (50.8)	49 (40.8)
High	44 (36.7)	54 (45.0)
Other	1 (0.8)	0 (0.0)
Unknown	3 (2.5)	4 (3.3)
Self-reported chronic illness (%)		
Yes	32 (26.7)	15 (12.5)
No	85 (70.8)	99 (82.5)
Unknown	3 (2.5)	6 (5.0)
Mean SF-12 Mental Component Summary score (SD)	46.3 (12.3)	50.2 (9.5)*
Mean SF-12 Physical Component Summary score (SD)	52.7 (7.6)	52.8 (7.3)
Mean distress thermometer score (SD)	3.4 (2.8)	2.6 (2.6)*

Children	
Child's sex (%)	
Male	60 (50.0)
Female	60 (50.0)
Diagnosis (%)	
Hematologic malignancy	42 (35.0)
Brain tumor	28 (23.3)
Solid tumor	42 (35.0)
Retinoblastoma	8 (6.7)
Relapse/second tumor (%)	
Yes	13 (10.8)
No	107 (89.2)
Mean child age at study in years (SD)	11.0 (5.7)
Mean time since diagnosis in months (SD)	39.4 (17.2)
Cumulative treatment^b^ (%)	
Low risk	26 (21.7)
Middle risk	53 (44.2)
High risk	41 (34.2)
Active treatment at time of study (%)	
Yes	16 (13.3)
No	104 (86.7)

^a^Defined according to Statistics Netherlands (CBS) [20]: low educational level = no education, primary school, lower secondary education; middle educational level = upper secondary education, preuniversity education, and intermediate vocational education; high educational level = higher vocational education, university. Other: foreign education

^b^Low risk therapy = no intervention, surgery only, local therapy other than radiation; middle risk therapy = chemotherapy with or without surgery; high risk therapy = any combination with radiation and/or stem cell transplant

*p < 0.05, indicating a significant difference between fathers and mothers

### Description of proxy-reported child HRQoL, parental QoL and distress

Compared to proxy reports of healthy children, fathers and mothers of children with cancer both reported significantly lower child HRQoL on all domains (Table 2).

**Table 2:** Proxy HRQoL ratings of mothers and fathers of children with cancer: differences with healthy children and level of parental agreement

Proxy-rated HRQoL scale	Parents of healthy children (*n* = 618–711) [21]	Fathers of children with cancer (*n* = 95–113)	Mothers of children with cancer (*n* = 95–113)	Parental agreement (oncology sample)	Mean difference father–mother (SD)	ES
	Mean (SD)	Mean (SD)	Mean (SD)	ICC [95% CI]		
Total HRQoL	87.6 (12.3)	80.1 (17.6)*	79.1 (18.0)*	0.83* [0.76, 0.88]	0.97 (10.4)	0.05
PSHS	86.6 (12.8)	79.2 (17.2)*	77.6 (17.8)*	0.78* [0.68, 0.85]	1.66 (11.7)	0.09
Physical functioning	89.3 (16.4)	80.9 (22.3)*	81.6 (22.2)*	0.83* [0.76, 0.88]	-0.68 (13.0)	0.03
Emotional functioning	82.6 (17.5)	74.4 (22.3)*	72.1 (21.9)*	0.80* [0.71, 0.86]	2.28 (14.0)	0.10
Social functioning	91.6 (14.2)	82.0 (18.6)*	80.4 (20.1)*	0.65* [0.53, 0.74]	1.64 (16.3)	0.08
School functioning	85.5 (17.6)	79.9 (20.2)*	78.0 (21.6)*	0.67* [0.54, 0.76]	1.95 (17.1)	0.09

*PSHS* Psychosocial Health Summary Score, *ES* effect size (Cohen’s *d*)

*p < 0.001; indicating a significant difference with parents of healthy children

Parent’s own QoL scores were available from 110 couples. Mothers reported lower mental QoL (MCS) scores than fathers (mean difference 3.9 ± 12.2, *p* = 0.001), but similar physical QoL (PCS) scores (Table 1). Additionally, mothers reported higher distress than fathers, although their average distress levels were not in the clinical range (mean thermometer score 3.4 vs 2.6, *p* = 0.005).

Table 3 shows the correlations between proxy-rated child HRQoL and parental QoL. In both fathers and mothers, small to moderate positive correlations (± 0.5) were found between parental MCS and parent-rated child’s total HRQoL score and psychosocial score (PSHS). Additionally, there was a moderate correlation between MCS in fathers and child’s emotional functioning. Correlations between parental MCS and other child domains were small (0.3–0.4). There were no significant correlations between maternal PCS and parent-rated child HRQoL. In fathers, own physical QoL (PCS) had a significant small correlation (0.3) with parent-rated child’s emotional functioning. Regarding distress, for both mothers and fathers, significant moderate negative correlations (0.4–0.6) were found between parental distress and all proxy scales.

**Table 3:** Correlations between maternal and paternal proxy HRQoL ratings and own QoL and distress

Proxy-rated HRQoL scale	MCS mothers	PCS mothers	MCS fathers	PCS fathers	Distress mothers	Distress fathers
Total HRQoL	0.47*	− 0.01	0.47*	0.18	− 0.53*	− 0.54*
PSHS	0.46*	0.04	0.47*	0.19	− 0.52*	− 0.56*
Physical functioning	0.38*	− 0.03	0.38*	0.14	− 0.46*	− 0.43*
Emotional functioning	0.35*	0.12	0.50*	0.26*	− 0.44*	− 0.61*
Social functioning	0.31*	0.11	0.41*	0.13	− 0.42*	− 0.37*
School functioning	0.42*	− 0.05	0.31*	0.07	− 0.39*	− 0.40*

*PSHS* Psychosocial Health Summary Score, *MCS* Mental Component Summary score, *PCS* Physical Component Summary score

*p < 0.001

### Agreement in paternal and maternal proxy HRQoL scores

Table 2 summarizes parental agreement on different PedsQL subscales. Differences in proxy ratings between fathers and mothers were not significant and effect sizes were small (0.03–0.10). Intra-class correlation coefficients were good on the PSHS, social functioning and school functioning scales, and excellent on the physical functioning, emotional functioning and total HRQoL scales.

**Fig. 1:**
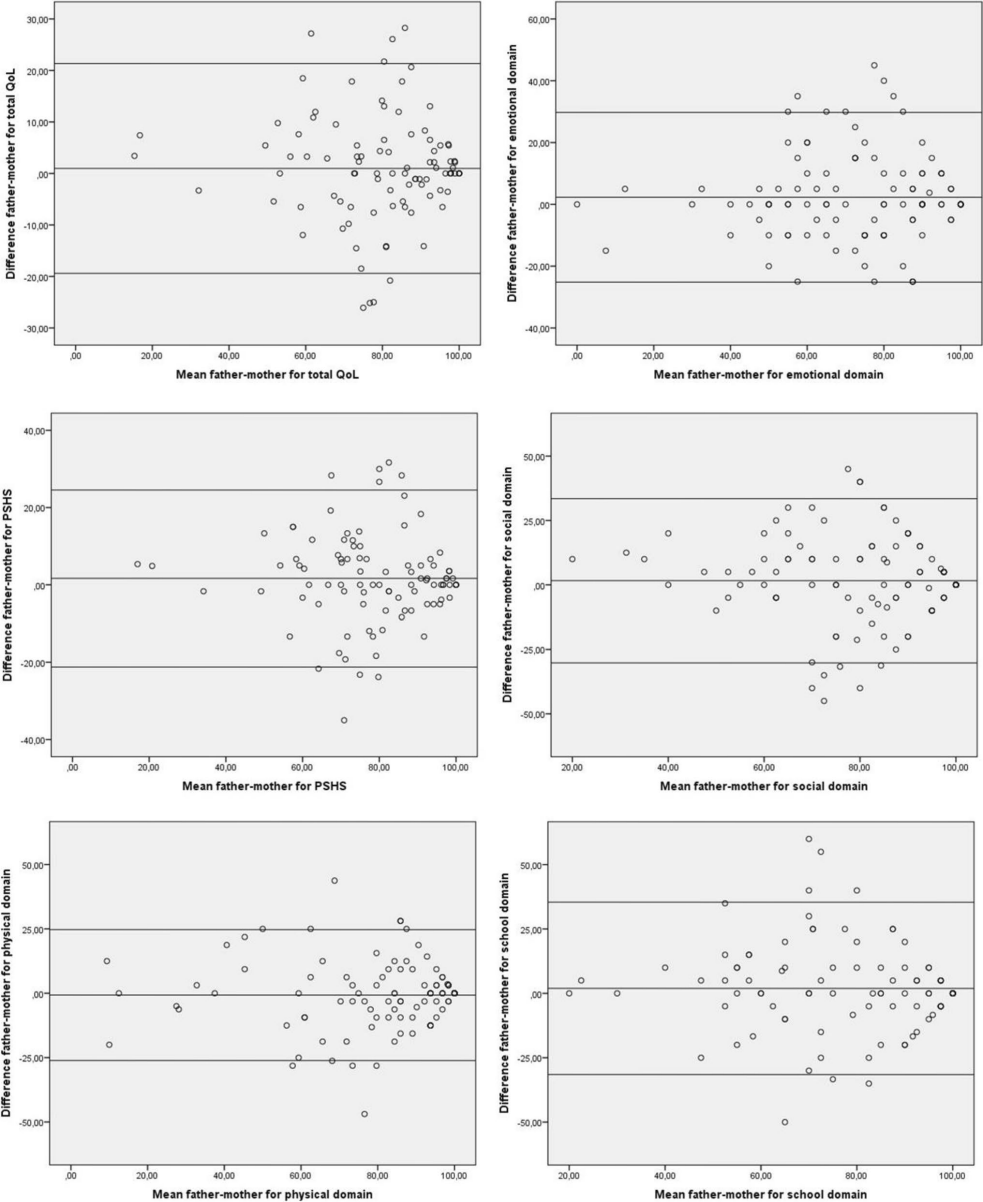
Bland–Altman plots for each proxy HRQoL scale, indicating patterns of disagreement between fathers and mothers. Explanation of the plots: the mean of the father’s and mother’s score is plotted on the *x*-axis against the mean difference between the both parents (father’s score minus mother’s score) on the *y*-axis. Additionally, the horizontal reference line closest to zero indicates the mean difference between fathers and mothers of the entire sample, and limits of agreements are added as horizontal reference lines at ± 2SD of this mean difference

Bland–Altman plots (Fig. 1) did not reveal any systematic patterns in disagreement, but there was a very wide range in the magnitude of differences (up to 50 points in some couples). These differences went in both directions; i.e. both higher ratings by fathers compared to mothers and vice versa.

### Determinants of caregiver disagreement

Table 4 shows the quartiles of the proxy difference irrespective of the direction, with corresponding means. The mean difference in the highest quartile ranged from 18.6 (total HRQoL) to 29.9 points (school functioning). Univariate regression models are displayed in Table 5, and the final multiple regression models in Table 6. For the total score and PSHS score, couples with the largest difference were more likely to have a child in active treatment (OR 3.1 [0.8–11.2], *p* = 0.091, and OR 4.2 [1.2–15.2], *p* = 0.030, respectively). For the physical functioning and school functioning score, couples were more likely to diverge in their mental QoL (OR 1.1 [1.0–1.1], *p* = 0.023, and OR 1.1 [1.0–1.1], *p* = 0.06, respectively). For the emotional functioning score, couples were more likely to diverge when they had a child with retinoblastoma (OR 9.1 [1.5–54.9], *p* = 0.02) or a child with a relapse or second tumor (OR 6.5 [1.8–22.9], *p* = 0.004).

**Table 4:** Quartiles of proxy differences (highest minus lowest score) and corresponding means

Quartile	Total HRQoL score	PSHS score	Physical functioning score	Emotional functioning score	Social functioning score	School functioning score
	Mean difference (SD)	Mean difference (SD)	Mean difference (SD)	Mean difference (SD)	Mean difference (SD)	Mean difference (SD)
p0-25	0.38 (0.53)	0.54 (0.78)	0.00 (0.00)	0.00 (0.00)	0.00 (0.00)	0.00 (0.00)
p25-50	3.14 (0.80)	3.80 (1.24)	3.33 (0.61)	4.94 (0.27)	5.26 (1.31)	5.00 (0.00)
p50-75	7.16 (1.72)	8.29 (2.26)	9.38 (2.46)	10.0 (0.00)	11.4 (2.26)	11.5 (2.79)
p75-100	18.6 (5.53)	20.1 (6.68)	23.6 (8.30)	23.2 (8.01)	28.0 (8.37)	29.9 (11.3)

*PSHS* Psychosocial Health Summary Score

**Table 5:** Univariate logistic regression models for proxy difference in the highest quartile

	Difference in total HRQoL (p75-100)	Difference in PSHS (p75-100)	Difference in physical functioning score (p75-100)	Difference in emotional functioning score (p75-100)	Difference in social functioning score (p75-100)	Difference in school functioning score (p75-100)
	OR [95% CI]	OR [95% CI]	OR [95% CI]	OR [95% CI]	OR [95% CI]	OR [95% CI]
Child (medical) variables						
Age	0.94 [0.85–1.03]	0.97 [0.89–1.06]	1.02 [0.94–1.10]	0.98 [0.91–1.06]	0.99 [0.92–1.07]	0.98 [0.90–1.07]
Female sex	0.92 [0.35–2.34]	1.39 [0.55–3.46]	0.62 [0.25–1.55]	0.74 [0.32–1.71]	1.78 [0.76–4.15]	0.90 [0.36–2.22]
Brain tumor versus hematologic malignancy	0.85 [0.24–3.04]	1.10 [0.32–3.74]	0.87 [0.27–2.83]	0.40 [0.11–1.44]	1.14 [0.38–3.41]	0.73 [0.23–2.39]
Solid tumor versus hematologic malignancy	0.56 [0.18–1.72]	0.66 [0.22–1.97]	0.61 [0.20–1.87]	0.70 [0.25–1.92]	0.76 [0.27–2.14]	0.26 [0.08–0.85]***
Retinoblastoma versus hematologic malignancy	2.56 [0.31–21.0]	1.70 [0.24–12.0]	1.16 [0.19–7.04]	6.27 [1.09–36.3]***	1.62 [0.33–8.06]	1.22 [0.18–8.36]
Relapse or second tumor	1.39 [0.33–5.89]	2.06 [0.53–8.02]	1.67 [0.47–5.98]	4.94 [1.47–16.6]***	1.27 [0.36–4.46]	1.33 [0.32–5.57]
Time since diagnosis	0.99 [0.96–1.01]	0.99 [0.96–1.01]	0.97 [0.94–1.00]***	1.00 [0.97–1.02]	0.99 [0.97–1.02]	1.00 [0.98–1.03]
Active treatment	3.06 [0.84–11.2]**	4.17 [1.15–15.2]***	3.67 [1.10–12.2]***	1.64 [0.49–5.46]	1.00 [0.30–3.44]	2.43 [0.70–8.49]**
Parental couples’ variables						
Difference in educational level	0.77 [0.29–2.01]	0.56 [0.21–1.49]	0.63 [0.24–1.66]	0.85 [0.36–2.03]	0.70 [0.29–1.65]	0.87 [0.34–2.22]
Difference in cultural background	–	–	0.57 [0.07–4.94]	0.39 [0.05–3.37]	1.11 [0.20–6.07]	–
Difference in mental quality of life (MCS)	1.02 [0.96–1.08]	1.03 [0.97–1.08]	1.06 [1.00–1.11]***	1.00 [0.95–1.05]	1.01 [0.97–1.06]	1.05 [1.00–1.11]**
Discrepancy in distress level (clinical/non clinical)	2.29 [0.79–6.60]*	1.79 [0.63–5.02]	2.69 [0.97–7.45]**	1.92 [0.76–4.86]	1.07 [0.41–2.79]	1.08 [0.37–3.19]

*PSHS* Psychosocial Health Summary Score, *MCS* Mental Component Summary score

*p < 0.15; **p < 0.10; ***p < 0.05

**Table 6:** Final multivariable logistic regression models for proxy difference in the highest quartile

	Difference in total HRQoL (p75-100)	Difference in PSHS (p75-100)	Difference in physical functioning score (p75-100)	Difference in emotional functioning score (p75-100)	Difference in school functioning score (p75-100)
	OR [95% CI]	OR [95% CI]	OR [95% CI]	OR [95% CI]	OR [95% CI]
Child (medical) variables					
Age					
Female sex					
Brain tumor versus hematologic malignancy				0.41 [0.11–1.60]	
Solid tumor versus hematologic malignancy				0.80 [0.27–2.34]	
Retinoblastoma versus hematologic malignancy				9.14 [1.5–54.9]**	
Relapse or second tumor				6.49 [1.84–22.9]**	
Time since diagnosis					
Active treatment	3.06 [0.84–11.2]*	4.17 [1.15–15.2]**			
Parental couples’ variables					
Difference in educational level					
Difference in cultural background					
Difference in mental quality of life (MCS)			1.06 [1.00–1.11]**		1.05 [1.00–1.11]*
Discrepancy in distress level (clinical/non clinical)					

*PSHS* Psychosocial Health Summary Score, *MCS* Mental Component Summary score

The Social Functioning domain is not displayed since no significant variables were retained in the final regression model

*p < 0.10; **p < 0.05

## Discussion

### Main findings and implications

The aims of this study were to assess potential differences in proxy HRQoL ratings between fathers and mothers of children with cancer, and to explore determinants of caregiver disagreement. On average, we found good agreement in paternal and maternal proxy ratings, and no significant differences between parents. This implies that if only one report is available, clinicians and researchers can reasonably assume that, in general, paternal and maternal proxy HRQoL ratings of their child with cancer are interchangeable. However, when comparing the highest rating parent to the lowest rating parent (instead of concentrating on the difference between fathers and mothers), proxy reports in the highest quartile differed on average 20 points. This means that approximately 25% of parents report quite differently about their child’s HRQoL. These couples were more likely to have a child in active treatment or with a relapse, have a child with retinoblastoma, and to diverge in their own mental QoL—compared to the parental couples with a proxy difference in the lower three quartiles (a difference between 0 and 10 points on most scales). Yet for some scales (e.g. social functioning) we could not specify determinants of this discrepancy.

This study adds important information regarding the interpretation of parent-reported outcomes in pediatric oncology.

Previous research in healthy children showed that paternal and maternal proxy HRQoL reports were interchangeable [12]. Moreover, in a sample of adolescent burn survivors as well as in children and adolescents in outpatient psychotherapy treatment, moderate to high agreement was found in proxy HRQoL reports of parent-dyads [30, 31]. However, to our knowledge, no previous studies have been performed on this subject in pediatric oncology. Furthermore, previous studies only focused on differences in paternal and maternal reports, and perhaps overlooked differences that would have been found if they had compared the highest and lowest proxy ratings. Also, determinants of caregiver disagreement have not been previously studied yet.

It is known that mothers are most often the primary caregivers. Especially in pediatric illness populations, mothers report higher distress and lower QoL, factors that can both influence proxy ratings [15–17]. Also in our study population, mothers reported lower QoL and higher distress for themselves than fathers. Therefore, if parents would rate their child’s HRQoL differently, we anticipated mothers to give lower ratings than fathers. This hypothesis is further strengthened by findings of previous studies in primary caregivers of healthy children, which showed that the relationship between parent’s own (mental) health and their proxy ratings was specifically present in mothers, more than in fathers [15, 18].

However, results of our study show that differences in proxy ratings were bidirectional, and in *both* parents equally correlated with own distress and mental QoL. The latter could explain why specifically the *difference* in mental QoL within a couple was a significant predictor of finding a large proxy HRQoL difference (for the physical functioning and school functioning scales). Furthermore, these findings might indicate that the situation of healthy children cannot be directly applied to pediatric illness populations, but future research is warranted.

We found that parents were more likely to differ in their proxy-rated total HRQoL and PSHS scores if their child was still in active treatment. This finding should be interpreted with caution, since we only had few children in our sample that were still in active treatment. Yet a previous study also found differences in agreement on and off treatment, although this study investigated child proxy agreement instead of parental agreement [19]. Especially *during* the child’s cancer treatment the primary caregiver is likely to be more closely involved with the child than the second caregiver [32, 33]. It might be that this could either enhance or relieve the worries of the second parent, considering the bidirectional differences that we found in the proxy ratings, but this warrants further research.

Additionally, for the emotional functioning score, we found larger differences if parents had a child with retinoblastoma or a relapse. This might be explained by the larger uncertainty that these characteristics entail; i.e. the hereditary nature of retinoblastoma or fear of blindness, and the worse prognosis of relapsed cancer.

Based on previous studies, we would have expected that a discrepancy in distress level between parents would be a significant predictor as well [15, 16]. However, we did not find this. An explanation might be that our sample did not have clinical distress scores on average.

With regards to sociodemographic factors, we did not find a relationship between having a proxy difference in the highest quartile and differences between parents in educational level or cultural background, although we know that these factors can influence proxy ratings [3, 4]. This is probably explained by the little variance that we had in our sample regarding these variables. The majority of parents in our sample had a Dutch background and middle or high educational level, which is not representative for the entire Dutch population.

Finally, age and sex of the child did not predict a larger proxy difference in our study. Previous studies found conflicting results regarding these factors [3]. It might be that parents with a very young child diverge more in their scores, since it is more difficult to rate for example emotional functioning if a child cannot well express his feelings [4]. However, the children in our study were on average 11 years old, and we had few toddlers in our sample (i.e. 17 children under the age of 4 years).

### Clinical implications

Several implications can be derived from our study. First, in some hospitals, electronic PROs are already systematically implemented in clinical practice. An example of this is the KLIK-portal in pediatric oncology [34, 35]. Before each outpatient visit—both during and after cancer treatment—parents and children from 8 years of age complete an online HRQoL assessment through this portal. A ten points decline in child’s HRQoL over time is flagged as clinically relevant. Since only one parent completes the proxy report and this parent may differ at various time points, our study findings are directly important to clinical care. About 25% of the parents in our study diverged more than 10 points, especially during treatment. In this case, if the respondent across both time points would be different, a decline in child’s HRQoL might reflect a proxy difference rather than a real decline. It should therefore be part of health-care providers’ training in using PROs in clinical practice that caregivers might differ in their proxy reports. This could be specifically important in divorced parents. Unfortunately, we had too few divorced parents in our sample to draw any conclusions on this group.

Furthermore, an important conclusion of our study is that parent gender is not really important in the interpretation of proxy reports; if in doubt about the interpretation, clinicians should be aware of the family context (i.e. child’s clinical characteristics and parents’ own functioning). Future studies should further explore the characteristics of the parents with a clinically relevant difference in proxy ratings. Additionally, it would be interesting to investigate parental agreement in other pediatric illness populations.

### Limitations

Our study has several limitations. First, the cross-sectional design only provides information on one specific moment in time, and the relatively low response rate might indicate some participation bias. Second, the far majority of the parent-dyads in our sample had a middle or high educational level, were born in the Netherlands, and were living together, which is not comparable to the general population. Especially in divorced parents, agreement in parents might be lower, but this should be confirmed in future research. Additionally, since there are no Dutch reference values available of the PedsQL proxy reports from 8 years of age, we used the reference values of Varni et al.; yet Dutch values might be slightly different. Furthermore, we did not have information on which parent was the primary caregiver; perhaps we would have found that this parent’s rating was consequently higher or lower than the second parent’s rating. However, we do not really expect this, since the mother is still the primary caregiver in the majority of the Dutch families [14]. Finally, it would have been interesting to include child self-reports as well, and to take these into account in the comparison of parental proxy ratings.

## Conclusions

We found few differences between paternal and maternal proxy reports of children’s HRQoL in pediatric cancer. This implicates that clinicians and researchers may reasonably assume that, in general, mothers’ and fathers’ reports are interchangeable. However, if possible, proxy reports of both parents should be included, since we have shown that 25% of parents differ widely in their scores. If only one report is available and there are any doubts of its interpretation, the respondent’s sex is not of major importance, but clinicians should instead be aware of the patient’s and family’s characteristics. Attention to these results is warranted in care, and future research should further explore the characteristics of these parents.
